# Suppression of Myocardial Injury Markers following Percutaneous Coronary Interventions by Pre-treatment with Carvedilol

**Published:** 2012-09-15

**Authors:** Abdolrasoul Moloudi, Feridoun Sabzi, Shirin Rashidi

**Affiliations:** 1 Emam Ali Cardiovascular Center, Kermanshah University of Medical Sciences, Kermanshah, IR Iran

**Keywords:** Carvedilol, Troponin I, CPK-MB, Coronary Interventions

## Abstract

**Background:**

Retrospective studies and clinical trials have indicated that β-receptor blockers have an influential role in improving survival and reducing risk of recurrent infarction in patients with myocardial infarction. However, there is still controversy regarding the effects of β-receptor blockers on the markers of myocardial infarction following percutaneous coronary interventions (PCI).

**Objective:**

The aim of this study was to evaluate the pre-treatment effect of Carvedilol on markers of myocardial injury in patients undergoing elective PCI.

**Method and Materials:**

In this clinical trial patients undergoing elective PCI were categorized randomly in the Carvedilol group including 100 patients who received two doses of 12.5 mg, 6 and 12 hours prior to PCI, and the control group (105 patients). Blood samples were obtained to analyse cardiac biomarker, 12 and 24 hours after PCI.

**Results:**

The clinical features were not significantly different between the two groups. A increase in the level of Troponin I was observed in the control group 24 hours following PCI (P=0.042), whereas this rise in troponin I was slight and insignificant in the Carvedilol group (P>0.05). some difference was observed between the two groups in regard to the level of CPK-MB after PCI (P=0.041).

**Conclusion:**

The findings of our study indicate that pre-treatment with Carvedilol confers cardio-protection by limiting the rise of markers of myocardial injury following PCI.

## Introduction

Percutaneous coronary intervention has been extensively used for treating ischemic heart disease, with success rate as high as 90% (1). Although acute and severe complications are rare with this method, a mild and asymptomatic release of biochemical markers of myocardial necrosis has sometimes been reported following (PCI) (2). Short episodes of myocardial ischemia occur during PCI which may result in myocardial injury by establishing a durable oxidative stress and augmenting lipid peroxidation and reducing antioxidant defense (3,4). Previous studies indicate that any slight rise in the creatine phospho kinase (CPK-MB) following a successful PCI renders the prognosis less favorable compared to patients without this enzyme rise (5,6). On the other hand, rise in level of Troponin I has been reported in 13.6-48% of patients after PCI and it has been demonstrated to be related to major clinical events such as re-infarction or sudden death during the patients’ follow-up (7,8). Thus, medical interventions prior to PCI may prove beneficial in improving the prognosis of these patients. Several trials conducted on secondary prevention have indicated that β-blockers reduce the risk of re-infarction and death following acute myocardial infarction (9,10). Data regarding the therapeutic effects of β-blockers on biochemical myocardial markers after successful PCI are controversial. Findings of an observational study indicated that using oral β-blocker prior to PCI reduced the level of CPK-MB and improved long-term survival (11). However, other studies did not corroborate these findings (12). On the other hand, the findings of a randomized trial indicated that administering Propranolol into the coronary arteries may protect the myocardial tissue during PCI and attenuate the rise in the level of Troponin I and CPK-MB (13). Carvedilol has been demonstrated to have further advantages compared to its preceding β-blockers which is due to its antioxidant effects alongside its nonspecific suppression of α and β receptors (14). Furthermore, it has been suggested that Carvedilol prevents lipid peroxidation in the membrane of myocardial cells and thus protects the endothelial, nervous and vascular muscular cells against the injuries caused by oxygen radicals (15). Laboratory data indicated that unlike the classical β-blockers, Carvedilol may induce coronary artery dilatation through nitric oxide (16). Based on these data, the present study was conducted with the purpose of studying the effects of pre-treatment with Carvedilol on serum levels of myocardial injury markers following percutaneous coronary interventions (PCI).

## Materials and Methods

This clinical study comprised Patients with coronary artery disease who underwent elective PCI in Emam Ali Hospital, Kermanshah. The exclusion criteria were myocardial infarction during the recent week, high levels of Troponin I and CPK-MB before PCI, severe left ventricle failure and contraindications of β-blockers.

The patients were categorized in two groups including 100 patients who received 12.5 mg Carvedilol orally the night before PCI and 12.5 mg Carvedilol orally in the morning PCI and control group without β-blockers. All patients received 600 mg Clopidogrel and 325 mg aspirin at least 6 hours prior to PCI. Blood samples were obtained before PCI and 12 and 24 hours after PCI.

The level of CPK-MB was measured by a new method for determination of CPK-MB (DGKC) method with an upper limit 24 IU/L. The level of Troponin I was measured using ELISA with an upper limit of 2.5 ng/mL.

## Statistical Analysis

Data obtained from both groups was analyzed using t-test and ANOVA (analysis of variance). In order to determine the difference between serum level of myocardial injury biomarkers, post-hoc analysis was used with Tukey’s test. For qualitative analysis of data, the χ2 test was used. In all cases, p<0.05 was considered significant.

## Results

A total of 205 patients entered our study, comprising 105 patients in the control and 100 in the Carvedilol group. The characteristics of patients prior to the intervention exhibited in Table 1 show no difference in demographic features between the two groups. Moreover, as demonstrated in Table 2 there is no significant difference between both groups in regard to cardiovascular risk factors (P=0.818).

**Table 1:** Demographic Features of Patients in Two Groups.

	Control (n=105)	Carvedilol (n=100)	P value
**Age**	55.5±9.5	56.7±10.5	0.402
**Gender (M/F)**	76/29	80/20	0.201
**BMI a**	25.4±4	25.9±3.5	0.326
**FBS a**	114±39.6	112.1±38	0.818
**TG a**	180±76	182±86	0.886
**Chol a**	186±60	180±44	0.453
**Cr**	0.97±0.13	0.96±0.15	0.364
**Hb a**	14.2±1.4	14.3±1.3	0.386
**Plt**	236±94	253±70	0.167
**LVEF a**	50±7.8	49±7.5	0.858
**Systolic BP a**	127±17	124±16	0.135
**Diastolic BP**	75±9	77±10	0.529
**Heart rate**	73±6.5	71.8±7.1	0.138

^a^Abbreviations: BMI, body mass index; FBS, fasting blood suger; TG, triglycerides; Chol, Cholesterol; Hb, hemoglobin; LVEF, left venticular ejection fraction; BP, blood perssure

**Table 2:** Cardiovascular Risk Factors in Two Groups

		Control (n=105)	Carvedilol (n=100)	*P* value
History of MI a (%)		36 (34)	45 (45)	0.117
History of HTN a (%)		58 (55)	44 (44)	0.108
History of CHF a (%)		24 (23)	22 (22)	0.883
History of Diabetes (%)		27 (26)	17 (17)	0.129
History of Smoking (%)	*Current*	17 (16)	19 (19)	0.860
	*Former*	37 (35)	35 (35)	
	*Never*	51 (48)	46 (46)	
History of PTCA a (%)		10 (9)	3 (3)	0.055
History of CABG a (%)		4 (4)	6 (6)	0.467

^a^Abbreviations: MI, myocardial infacrtion; HTN, Hypertension; CHF, congestive heart failure; PTCA, percutaneous coronary artery intervention; CABG, coronary artery bypass graft

The data in Table 3 relate to the process of PCI in which 15% of patients in the control group and 14% of those in the Carvedilol group had involvement of multiple coronary arteries. However, other patients , no significant difference found between the two groups in terms of features of coronary involvement and the process conducted on the coronary arteries.

**Table 3:** Data Concerning the Performance Of Pci in Two Groups

	Control (n=105)	Carvedilol (n=100)	P value
**Multi- vessel disease**	16 (15%)	14 (14%)	0.802
**Minor branches**	5 (4%)	6 (6%)	0.694
**Major branches**	101 (96%)	93 (93%)	0.311
**Stent use**	104 (99%)	98 (98%)	0.532
**Number of stent used (Average for each patient)**	146 (1.4)	134 (1.36)	0.427

## Laboratory Evaluation of Markers of Myocardial Injury

As depicted in Table 4, rises in serum level of Troponin I were observed at 12 and 24 hours following PCI in both control and Carvedilol groups. This rise, however, was milder in patients receiving Carvedilol prior to PCI. Thus, in the control group, there was a significant decrease in the level of Troponin I at 24 hours after PCI compared to its level before the intervention, whereas in the Carvedilol group, there was significant increase in serum level of CPK- MB after 12 hours in the control group with no significant difference in both groups after 24 hours (P=0.041).

**Table 4:** Biochemical Markers of Myocardial Injury before and After PCI in Two Groups

	Control (n=105)	Carvedilol (n=100)	P value
**TnI a (Before)**	0.24±0.33	0.24±0.39	0.897
**TnI (After 12h)**	0.46±0.8	0.43±0.93	0.820
**TnI (after 24h)**	0.57±1	0.45±1.0	0.042
**CPK-MB (Before)**	17.53±3.7	17.76±3.6	0.661
**CPK-MB a (After 12h)**	31.6±7.1	19.4±6.1	0.041
**CPK-MB (After 24h)**	20.6±11.2	19.8±11.9	0.620

^a^Abbreviations: CPK-MB, creatine phospho kinase; TnI, Troponin I

## Discussion

Our findings indicate that the serum level of Troponin I increases considerably after PCI in patients who undergo elective PCI. This observation may suggest some degrees of myocardial injury during PCI, as reported in some previous studies(1). A study by Cavallini et al. on 3494 patients, who had undergone PCI, indicated that the level of CPK-MB rises in 16% of patients after PCI which was significantly related to their mortality during 2 years of follow-up (1). According to the results of a study performed by Riccardi et al. who had used MRI, the rise of serum levels of CPK-MB was due to identifiable myocardial necrosis (17). In another study by Okmen et al., it was observed that the level of Troponin I rose in almost one third of patients who had undergone PCI successfully (18). One study conducted on 286 patients in 2003 reported increased levels of CPK-MB and Troponin I in 12.9% and 13.6% of patientsrespectively (19). The findings of a study by Nageh et al. on 109 patients showed rising levels of Troponin I in 58, Troponin T in 38, and CPK-MB in 28 patients after PCI. This rise in enzyme level was directly related to major cardiac events in the future (20).

Many other studies have attributed the elevated CPK-MB level after PCI to increased fatality, myocardial infarction and repeat revascularization (5,6,21). Moreover, studies by Adams et al (22) and Genser et al (23) related the rising levels of Troponin I after PCI to higher incidence of major cardiac events during the follow-up period. Similar results were reported in studies by Garbaz et al (24) and Cantor et al (7) concerning the elevated level of Troponin I after PCI. Therefore, it is evident that interventions which reduce the myocardial injury bio markers after PCI may improve the long-term outcome of PCI.

In our study, administering 12.5 mg Carvedilol prior to PCI prevented the rise of Troponin I after the process considerably, with its effect most pronounced at 24 hours after PCI. Few studies have been conducted on the effects of β-blockers on the biochemical myocardial markers after PCI with controversial results. Sharma et al. studied 1675 patients who had undergone PCI to indicate that pre-treatment with β-blockers has protective effects on heart during ,may reduce the levels of CPK-MB after, as well as improving the patients’ survival after PCI (11). Wang et al. reported that administering Propranolol into the coronary arteries during PCI reduced the increasing levels of Troponin I and CPK-MB by 20% and 19%, respectively, compared to the control group (13). In contrast, Ellis et al, reported that administering β-blockers prior to PCI had no effect on the increasing levels of CPK-MB after intervention (12). Also, Atar et al. administered 100 mg Metoprolol before PCI to conclude that it had no effect on limiting the increased level of Troponin I after PCI (8).

Numerous studies reported Carvedilol to be superior to other β-blockers. In a study by Nagatomo et al., Carvedilol ,unlike Metoprolol, was observed to attenuate the increased level of CRP in patients with congestive heart failure. Furthermore, contrary to Metoprolol, Carvedilol managed to reduce the lipid peroxidase in these patients (25). Also, Kastratovic et al. reported that Carvedilol increased the activity of copper-zinc superoxide dismutase in patients with acute myocardial infarction, whereas Metoprolol did not manifest this property (26). In another study on the effects of Carvedilol and Propranolol on oxidative stress in mononuclear and polynuclear cells in patients with hypertension, Carvedilol was reportedly much superior to Propranolol in inhibiting oxidative stress and reducing the level of CRP (27). All these studies pointed to the antioxidant effects of Carvedilol, as other researchers reported the antioxidant and cardiac protective properties of Carvedilol (28, 29). Kozlovski et al. found that unlike Atenolol and Labetalol, Carvedilol can dilate coronary arteries through inducing nitric oxide (16). Remme et al. studied 3029 patients with heart failure in 2007 and showed that Carvedilol was much more efficient than Metoprolol in preventing cardiovascular disorders(30).

The findings of our study indicate that administering Carvedilol prior to PCI may have beneficial effects in protecting the myocardium and preventing the rise of myocardial injury biomarker following PCI.
